# Decreased Serum *microRNA-21*, *microRNA-25*, *microRNA-146a*, and *microRNA-181a* in Autoimmune Diabetes: Potential Biomarkers for Diagnosis and Possible Involvement in Pathogenesis

**DOI:** 10.1155/2019/8406438

**Published:** 2019-09-09

**Authors:** Yiwen Liu, Minglei Ma, Jie Yu, Fan Ping, Huabing Zhang, Wei Li, Lingling Xu, Yuxiu Li

**Affiliations:** Department of Endocrinology, Key Laboratory of Endocrinology, Ministry of Health, Peking Union Medical College Hospital, Peking Union Medical College, Chinese Academy of Medical Sciences, Beijing 100730, China

## Abstract

**Objective:**

Previous studies have revealed dysregulated circulating microRNAs (miRNAs) in patients with type 1 diabetes (T1D). Here, we explored the serum levels of *miR-21*, *miR-25*, *miR-146a*, and *miR-181a* in patients with autoimmune diabetes (T1D and latent autoimmune diabetes of adults (LADA)) compared with type 2 diabetes (T2D) and nondiabetic individuals.

**Design, patients, and measurements:**

The serum levels of *miR-21*, *miR-25*, *miR-146a*, and *miR-181a* in patients with T1D (*n* = 29), LADA (*n* = 16), and T2D (*n* = 31) and in nondiabetic individuals (*n* = 19) were determined by quantitative real-time polymerase chain reaction, and receiver-operating characteristic (ROC) curves were evaluated to determine the discriminatory performances of these four miRNAs. Furthermore, target genes and pathways potentially modulated by these four miRNAs were predicted by bioinformatics analysis to investigate the possible functions of these miRNAs in autoimmune diabetes. Subsequently, multiple logistic regression analysis was performed to identify independent predictors for autoimmune diabetes, and a nomogram was established.

**Results:**

*miR-21*, *miR-25*, *miR-146a*, and *miR-181a* were significantly downregulated in the serum of patients with autoimmune diabetes compared with those in T2D patients and nondiabetic individuals (*p* < 0.001). The areas under the ROC curves of these four miRNAs were greater than 0.80 (*p* < 0.001). Bioinformatics analysis suggested that *miR-21*, *miR-25*, *miR-146a*, and *miR-181a* regulated multiple genes in pathways associated with immunity, inflammatory responses, hyperglycemia, and metabolism, which are involved in the pathogenesis of autoimmune diabetes. Multiple logistic regression analysis identified *miR-25* (odds ratio (OR): 0.001, *p* < 0.05), *miR-146a* (OR: 0.136, *p* < 0.05), and fasting C-peptide levels (OR: 0.064, *p* < 0.05) as independent predictors of autoimmune diabetes.

**Conclusions:**

*miR-25* and *miR-146a* may serve as potential circulating biomarkers and provide insights into the pathogenesis of autoimmune diabetes.

## 1. Introduction

Type 1 diabetes (T1D) is a chronic progressive autoimmune disease characterized by T-cell-mediated pancreatic *β*-cell destruction, ultimately leading to absolute insulin deficiency and exogenous insulin-dependent hyperglycemia [1]. Latent autoimmune diabetes of adults (LADA), another specific subtype of autoimmune diabetes with less intensive pancreatic *β*-cell destruction compared with T1D at its initial stage, shares several common immunological and genetic features with T1D, such as the existence of islet autoantibodies, the important role of cellular immunity in disease pathogenesis, and shared risk loci [2]. Despite much effort, autoimmune diabetes still faces enormous challenges, including delayed diagnosis owing to the overlap of clinical characteristics with type 2 diabetes (T2D), insufficient reliable biomarkers, lack of appropriate targets, and lack of strategies to reverse or delay islet *β*-cell failure, given the unclear mechanism. Therefore, biomarkers with the potential to facilitate diagnosis and provide insights into the pathogenesis of autoimmune diabetes are urgently needed.

MicroRNAs (miRNAs) are endogenous, evolutionarily conserved, small, double-stranded, noncoding RNAs measuring approximately 19–24 nucleotides in length; these molecules act as negative posttranscriptional modulators of mRNA expression [3]. Accumulating evidence indicates that multiple circulating miRNAs are dysregulated in T1D and participate in the pathogenesis of this disease, thus serving as potential noninvasive biomarkers and therapeutic targets for T1D. For example, *miR-21*, *miR-25*, *miR-146a*, and *miR-181a* [4] are associated with the regulation of immune responses, *β*-cell apoptosis and proliferation, and insulin biosynthesis and secretion, which are all involved in the pathogenesis of autoimmune diabetes. However, the roles of these miRNAs remain to be elucidated. *miR-146a* has essential regulatory roles in T-cell biology; however, most studies have used peripheral blood mononuclear cells (PBMCs) or T cells as samples, necessitating extended storage and processing before measurements. Moreover, few studies have evaluated serum *miR-146a* expression patterns in samples from patients with T1D. *miR-21*, which was previously reported to have critical roles in T-cell activation, is upregulated in serum from patients with T1D and speculated to have dual effects on pancreatic *β* cells [5, 6]; therefore, *miR-21* may dynamically change during different stages of T1D. *miR-25* has previously been reported to have prognostic value with regard to the functions of residual *β* cells and glycemic control several months later in patients with T1D [7], necessitating additional studies to explore its association with residual *β*-cell function and glycemic control. Moreover, *miR-181a* has been reported to play a prominent role in T-cell activation, which is important in the pathogenesis of T1D [8]. Furthermore, few studies have investigated alterations in these four miRNAs in the circulation of patients with LADA, another important subtype of autoimmune diabetes.

Accordingly, in this study, we examined alterations in the levels of *miR-21*, *miR-25*, *miR-146a*, and *miR-181a* in the serum of patients with T1D and LADA to identify potential circulating biomarkers and gain insights into the pathogenesis of autoimmune diabetes.

## 2. Materials and Methods

### 2.1. Study Populations

Using protocols and consent procedures approved by the ethics committee of the Peking Union Medical College Hospital, 95 individuals attending the Peking Union Medical College Hospital from January 2014 to May 2016 were recruited to the current study, including patients with T1D (*n* = 29), LADA (*n* = 16), and T2D (*n* = 31) and nondiabetic individuals (*n* = 19). All participants provided written informed consent for the collection of blood samples. Blood samples were collected and centrifuged for 10 minutes at 3000  rpm to separate serum, which was subsequently refrigerated at −80°C before further analysis. Diabetes was diagnosed based on the World Health Organization criteria [9]. The classification of T1D was made according to the characteristics as follows: dependence on prompt insulin treatment at a rapid onset of diabetes, fasting C-peptide or 2-h postprandial C-peptide lower than 0.8 ng/mL, and positivity for islet autoantibodies. The diagnostic criteria of LADA were as follows: 30–70 years old at diagnosis; positivity of one of the islet autoantibodies, including glutamic acid decarboxylase antibody (GADA), islet cell antibody (ICA), and protein tyrosine phosphatase antibody (IA-2A); and independence from insulin therapy for at least 6 months after diagnosis [10]. Exclusion criteria included suspected other types of diabetes and possibility of organ dysfunction, infection, and pregnancy. All patients with T1D or LADA were treated with insulin, and some patients also received oral antidiabetic agents, including metformin (T1D: 7/29, LADA: 5/16) and *α*-glycosidase inhibitors (T1D: 5/29, LADA: 6/16). T2D patients also received oral antidiabetic agents, including metformin (22/31), *α*-glycosidase inhibitors (24/31), dipeptidyl peptidase inhibitors (10/31), and sulfonylureas (13/31); few of these patients were treated with insulin (7/31). Demographic data were collected, including sex, age, age at diagnosis, diabetes duration, weight, height, and body mass index (BMI).

### 2.2. Biochemical Determinations of Study Populations

Biochemical determinations included glycosylated hemoglobin (HbA1c), total cholesterol (TC), total triglyceride (TG), high-density lipoprotein cholesterol (HDL-C), low-density lipoprotein cholesterol (LDL-C), fasting and 2-h postprandial plasma glucose (FPG and 2hPG), fasting and 2-h postprandial C-peptide (FCP and 2hCP), GADA, ICA, and IA-2A. HbA1c was determined by high-performance liquid chromatography. TG, TC, HDL-C, and LDL-C were measured using the oxidase method. Serum C-peptide levels were determined by chemiluminescent immunoassay (C-peptide, 03649928 (129026); Siemens, USA). The lower limit of detection was 0.05 ng/mL, and all undetected levels were reported as 0 ng/mL. GADA and IA-2A were measured by enzyme-linked immunosorbent assay (EA1022 and EA1023; Euroimmun, China). ICA was measured using an immunofluorescence method (FA1020; Euroimmun). The lowest detection limits for GADA, IA-2A, and ICA were 20, 20, and 10 IU/mL, respectively.

### 2.3. RNA Isolation and Reverse Transcription-Quantitative Polymerase Chain Reaction (RT-qPCR)

RNA isolation was performed on a total of 95 serum samples from the participants. Total RNA was extracted from 200 *μ*L serum with a miRcute miRNA Isolation Kit (miRNeasy serum/plasma kit; Qiagen, Valencia, CA, USA) according to the manufacturer's instructions. Reverse transcription was performed with a Takara SYBR PrimeScript miRNA RT-PCR Kit (MIR-x miRNA First-strand synthesis kit; Clontech). The primers specific for mature miRNA targeted *hsa-miR-21-5p*, *hsa-miR-25-3p*, *hsa-miR-146a-5p*, and *hsa-miR-181a-5p*. All samples were normalized to *hsa-miR-16* expression as a control. The primer sequences for *hsa-miR-21-5p*, *hsa-miR-25-3p*, *hsa-miR-146a-5p*, *hsa-miR-181a-5p*, and *hsa-miR-16* are listed in Supplementary [Supplementary-material supplementary-material-1]. RT-qPCR was performed with a Takara SYBR PrimeScript miRNA RT-PCR Kit (SYBR Premix Ex Taq II; Takara, Shiga, Japan). The reaction was run on an AB Real-Time PCR System (7900HT fast Fluorescent Quantitative PCR; ABI), and data were evaluated using the 2^−ΔΔCT^ method [11].

### 2.4. miRNA Target Gene Prediction and Pathway Analysis

Target gene prediction for these four miRNAs was performed using four web-based prediction tools, including MiRWALK [12], miRTarBase [13], miRDB [14], and TargetScan [15]. To control the false-positive rate, target genes were selected on the basis of at least three adopted prediction tools. Subsequently, functional enrichment analysis of target genes for these four miRNAs was performed with pathway annotations from the Kyoto Encyclopedia of Genes and Genomes (KEGG) database [16]. Significantly targeted pathways enriched for target genes were identified based on Fisher exact tests (*p* < 0.01).

### 2.5. Statistical Analysis

All analyses were implemented using SPSS Statistics software (Version 25.0; SPSS, Chicago, IL, USA), R (Version 3.5.0), and GraphPad Prism 6.0 (http://www.graphpad.com). Two-sided tests were used, and statistical significance was established at a *P* value of 0.05. Continuous data were presented as means ± standard deviations. Normal distributions were evaluated by Kolmogorow–Smirnow and Shapiro–Wilk tests. Differences between groups were tested by nonparametric Mann–Whitney or Kruskal–Wallis tests. Receiver-operating characteristic (ROC) curves were established, and the areas under the ROC curves (AUC-ROCs) were calculated to evaluate the discriminatory power of the four miRNAs to distinguish T1D and LADA from T2D and nondiabetic individuals. Spearman correlations were conducted to determine the associations among these four miRNAs and clinical variables. Multiple logistic regression analysis was performed to identify independent predictors for autoimmune diabetes, and a nomogram was constructed based on the regression coefficients (*β*) from the multivariate logistic model.

## 3. Results

### 3.1. Clinical Characteristics of Study Populations

In total, 95 participants were studied. As indicated in Table 1, patients with T1D were younger and had a younger age of diabetes onset compared with patients with LADA and T2D. No significant differences were found among the three diabetic groups with regard to diabetes duration and BMI. Patients with autoimmune diabetes (T1D and LADA) suffered from less favorable glycemic control and residual *β*-cell function, with significantly elevated islet autoantibody titers compared with patients with T2D.

### 3.2. Serum *miR-21*, *miR-25*, *miR-146a*, and *miR-181a* Were Downregulated in Autoimmune Diabetes

**Figure 1:**
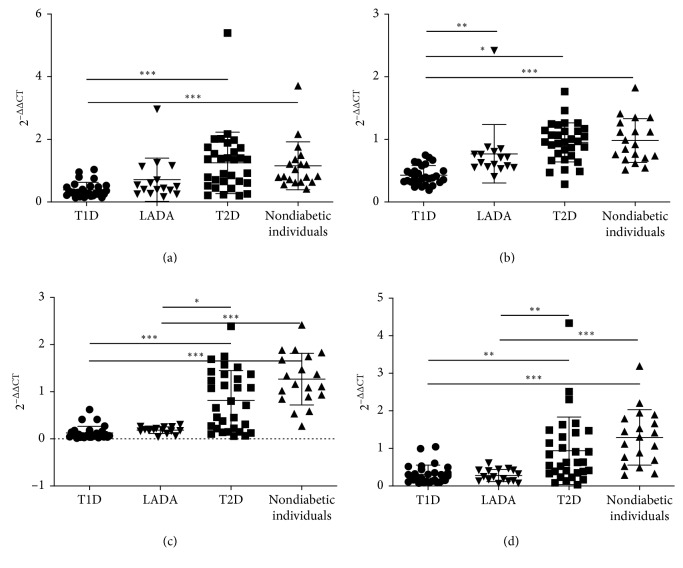
Serum levels of *miR-21*, *miR-25*, *miR-146a*, and *miR-181a* in T1D, LADA, T2D, and nondiabetic individuals. (a) miRNA-21. (b) miRNA-25. (c) miRNA-146a. (d) miRNA-181a. ^*∗*^*p* < 0.05; ^*∗∗*^*p* < 0.01; ^*∗∗∗*^*p* < 0.001; 2^−ΔΔCT^ represents serum levels of miRNAs. Abbreviations: T1D, type 1 diabetes; LADA, latent autoimmune diabetes; T2D, type 2 diabetes.

Differences in the serum levels of these four miRNAs were not significant between T2D patients and nondiabetic individuals. In contrast, the levels of these four miRNAs were downregulated in patients with T1D and LADA compared with those in patients with T2D and nondiabetic individuals, although the difference for patients with LADA was not significant. Additionally, decreases in the levels of these four miRNAs in individuals with LADA were weaker than those in patients with T1D, consistent with the notion that LADA is an intermediate form between T1D and T2D (Figure 1).

**Figure 2:**
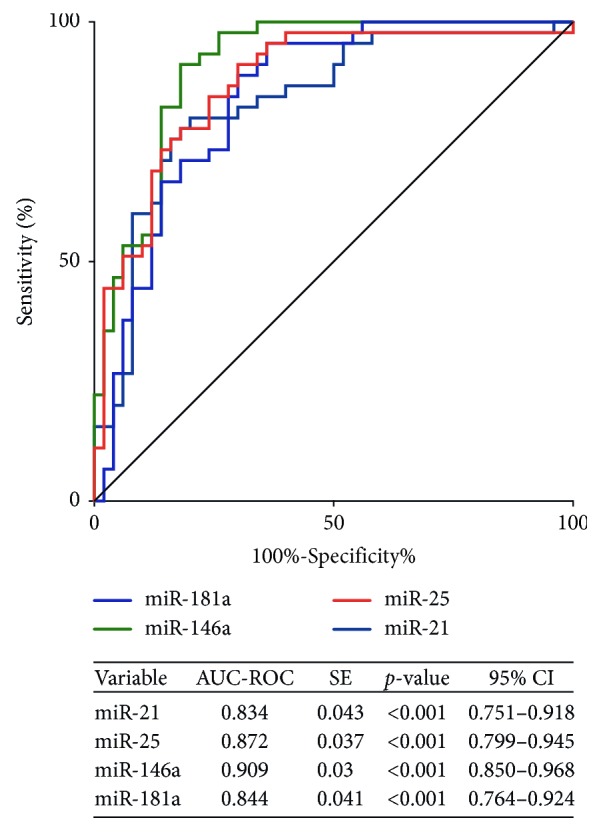
Receiver-operating characteristic curves for discriminatory performance of *miR-21*, *miR-25*, *miR-146a*, and *miR-181a* in distinguishing T1D and LADA from T2D and nondiabetic individuals. Abbreviations: SE, standard error; 95% CI, 95% confidence interval; T1D, type 1 diabetes; LADA, latent autoimmune diabetes; T2D, type 2 diabetes.

Next, ROC curves were performed to evaluate the discriminatory performance of the four miRNAs in distinguishing T1D and LADA from T2D and nondiabetic individuals. As shown in Figure 2, AUCs of the four miRNAs were all greater than 0.8 (*miR-21*: 0.834, 95% confidence interval (CI): 0.751–0.918; *miR-25*: 0.872, 95% CI: 0.799–0.945; *miR-146a*: 0.909, 95% CI: 0.850–0.968; *miR-181a*: 0.844, 95% CI: 0.764–0.924), indicating the favorable performance of these miRNAs for distinguishing T1D and LADA from T2D and nondiabetic individuals and their potential to serve as circulating biomarkers for autoimmune diabetes.

### 3.3. Serum Levels of *miR-21*, *miR-25*, *miR-146a*, and *miR-181a* Did Not Correlate with Glycemic Control, Residual *β*-Cell Function, and Islet Autoantibodies in Autoimmune Diabetes

Spearman correlation analysis was conducted to explore the correlations of serum levels of *miR-21*, *miR-25*, *miR-146a*, and *miR-181a* with clinical parameters in autoimmune diabetes (Table 2). Notably, these miRNAs showed no correlations with HbA1c, FCP, or 2hCP, indicating no associations between serum levels of these miRNAs and glycemic control or residual *β*-cell function. Additionally, we failed to identify associations between serum levels of these miRNAs and titers or numbers of positive islet autoantibodies. Nonetheless, serum levels of *miR-25* were positively correlated with age (correlation coefficient: 0.576, *p* < 0.01), BMI (correlation coefficient: 0.296, *p* < 0.05), and age of diabetes onset (correlation coefficient: 0.483, *p* < 0.01).

### 3.4. Target Genes and Pathway Predictions for *miR-21, miR-25, miR-146a,* and *miR-181a*

**Figure 3:**
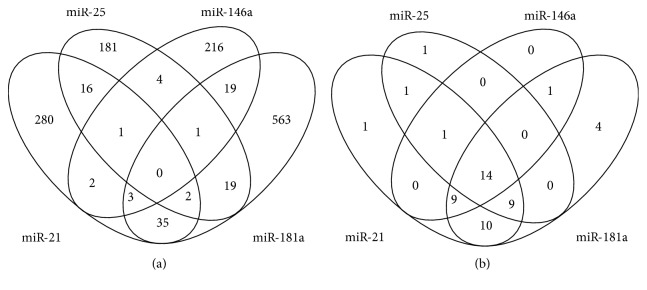
Venn diagram of predicted target genes and KEGG pathways for *miR-21*, *miR-25*, *miR-146a*, and *miR-181a*. (a) Venn diagram of predicted target genes. The genes were selected as targets if they had overlapped in at least three of four prediction algorithms (MiRWALK, miRTarBase, miRDB, and TargetScan). (b) Venn diagram of KEGG pathways enriched with genes targeted by the four miRNAs with *p* < 0.01. Pathways related to malignancy were eliminated. Abbreviations: KEGG, Kyoto Encyclopedia of Genes and Genomes.

To investigate the possible functions of these four miRNAs in autoimmune diabetes, bioinformatics analysis was implemented to predict target genes and pathways potentially affected by these miRNAs. In total, 1,481 genes were predicted by at least three prediction tools for these four miRNAs. Subsequently, a multitude of genes were found to be targeted by multiple miRNAs; after excluding duplicates, 1,342 unique genes were predicted to be targeted by these four miRNAs (Figure 3(a)). Forty-five, 26, 25, and 47 pathways were identified as enriched with genes targeted by *miR-21*, *miR-25*, *miR-146a*, and *miR-181a*, respectively, after excluding malignancy-associated pathways. Some pathways overlapped, yielding 51 unique pathways. There were 14 pathways enriched with genes targeted by the four miRNAs, among which the most statistically significant pathway was the tumor necrosis factor (TNF) signaling pathway (*p* < 1.54 × 10^−4^), and other pathways (*p* < 0.001) included the AGE-RAGE signaling pathway in diabetic complications, mammalian target of rapamycin, sphingolipid, Chagas disease, influenza A, Toll-like receptor, and T2DM signaling pathways. Pathways with a statistical significance of *p* < 0.01 included the RIG-I-like receptor, cGMP-PKG, insulin resistance, tight junction, hepatitis B, and Epstein-Barr virus infection signaling pathways. Additionally, 19 pathways were enriched with three miRNAs, among which the mitogen-activated protein kinase (MAPK) signaling pathway (*p* < 5.17 × 10^−7^) and neurotrophin signaling pathway (*p* < 3.14 × 10^−6^) were the most significant (Supplemental [Supplementary-material supplementary-material-1]).

### 3.5. *miR-25*, *miR-146a*, and FCP May Serve as Independent Predictors for Autoimmune Diabetes

Using multiple logistic regression analysis, *miR-25* (OR: 0.136, 95% CI: 0.020–0.931; *p*=0.042), *miR-146a* (OR: 0.001, 95% CI: 0.001–0.219; *p*=0.011), and FCP (OR: 0.064, 95% CI: 0.014–0.288; *p*=0.001) were identified as independent predictors for autoimmune diabetes (Supplemental [Supplementary-material supplementary-material-1]).

**Figure 4:**
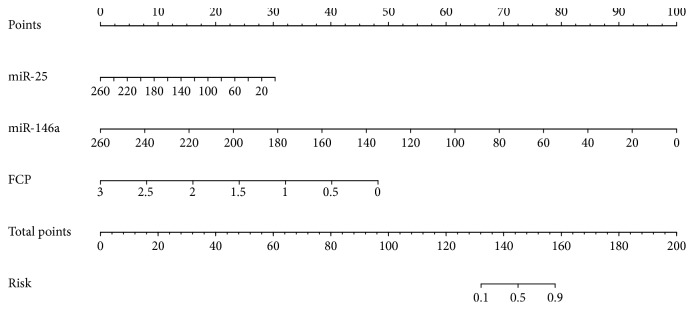
Nomogram to estimate the risk of autoimmune diabetes. To utilize the nomogram, seek out the value of each variable on the corresponding axis, mark a line to extend to the points axis to obtain the points, add the points of all variables together, and draw a line from the Total points axis to the Risk axis to determine the probability of autoimmune diabetes. Abbreviations: FCP, fasting C-peptide.

A nomogram was constructed based on these independent predictors (Figure 4), with favorable predictive performance in internal validation measured by ROC curves (AUC: 0.968, 95% CI: 0.922–0.989, sensitivity: 88.7%, and specificity: 94.2% at the optimal cut-off score).

## 4. Discussion

Reliable biomarkers are required to achieve more accurate diagnosis and provide insights into the regulation of the pathogenesis of autoimmune diabetes. In this study, we demonstrated significant downregulation of serum *miR-21*, *miR-25*, *miR-146a*, and *miR-181a* in T1D and LADA patients compared with T2D patients and nondiabetic individuals. Bioinformatics analysis revealed that these four miRNAs targeted 1,342 genes, which could impact 51 KEGG pathways, most of which were related to immunity, inflammation responses, diabetes, and metabolism and were therefore involved in autoimmune diabetes. *miR-25*, *miR-146a*, and FCP were independent predictors of autoimmune diabetes, suggesting their potential to serve as circulating biomarkers of autoimmune diabetes, which may be of immense value in clinical practice.

Several previous studies reported increased *miR-21* in the plasma/serum of patients with T1D compared with that in nondiabetic individuals, whereas one study found no differences in *miR-21* expression in the plasma between patients with T1D and patients with T2D [7, 17–19]. In contrast, we found a significant decrease in *miR-21* levels in the serum of patients with T1D compared with that in nondiabetic individuals and patients with T2D in this study. This difference could be related to differences in blood samples or clinical features of the participants included in the different studies (e.g., sex, age, and disease duration). *miR-21* has previously been identified as a critical regulator of immune responses, participating in the regulation of anti-inflammatory responses [20], T lymphocyte activation [21], and regulatory T-cell (Treg) development [22]. Additionally, a growing number of studies have indicated the dual regulatory effects of *miR-21* in pancreatic *β*-cell apoptosis, including antiapoptotic effects via suppression of tumor-suppressive programmed cell death [4], which promotes cell death [5] and pro-apoptosis effect via inhibition of the antiapoptotic BCL2 [6]. Additionally, *miR-21* is associated with impaired glucose-stimulated insulin secretion [23].

Changes in circulating *miR-25* levels reported in previous studies have not been conclusive. One publication showed downregulation [18], whereas others showed upregulation of *miR-25* in the serum/plasma of patients with T1D compared with that in nondiabetic individuals [7, 11, 24]. Notably, serum *miR-25* measured 1 month after T1D onset was positively correlated with stimulated C-peptide levels and negatively correlated with HbA1c measured 3 months after T1D onset, suggesting its potential prognostic role in predicting *β*-cell functions and glycemic control in the progression of T1D [7]. However, we failed to find an association between serum *miR-25* and glycemic control or residual *β*-cell function, probably resulting from the different disease course between the study populations different analytical platforms applied and the absence of follow-up data after diabetes onset in the current study. Several previous publications have shown that *miR-25* has multiple effects on the regulation of apoptosis via multiple pathways in various types of cancer [25, 26]. However, previous studies have rarely investigated the associations between *miR-25* and pancreatic *β*-cell apoptosis in T1D.

Similarly, alterations in *miR-146a* in different studies have also been contradictory. *miR-146a* was previously reported to be reduced in PBMCs of patients with T1D [11, 27], whereas *miR-146a* was increased in T lymphocytes of patients with T1D in another publication [28]. *miR-146a* is essential for the immunosuppressive function of Tregs and is thus recognized as a canonical regulator of immune responses [29]. Loss of *miR-146a* could generate spontaneous autoimmunity. Interestingly, a previous study showed that decreased *miR-146a* in PBMCs was correlated with high GADA titers and GADA positivity, supporting that *miR-146a* may be associated with the ongoing islet autoimmunity in T1D [11, 27]; however, we did not replicate this observation. Interestingly, despite their important roles as biomarkers of autoimmunity, islet autoantibodies do not have major roles in pancreatic *β*-cell destruction.

In previous studies, *miR-181a* was found to be upregulated in the serum of patients with T1D [7, 30]. However, in this study, we found the opposite results. Moreover, loss of *miR-181a* increases the reactivity of peripheral T cells against self-antigens, indicating its essential role in modulation of T-cell activity [8]. *miR-181a* also has anti-inflammatory effects given its ability to inhibit increases in TNF, interleukin (IL)-1*β*, and IL-6 in macrophages treated with lipopolysaccharides [31].

In the current study, we found markedly decreased levels of serum *miR-21*, *miR-25*, *miR-146a*, and *miR-181a* in autoimmune diabetes, inconsistent with some previous studies. This discrepancy could be attributed to differences in blood samples collected, detection methods of miRNAs, and clinical features of the participants (e.g., sex, age, and disease duration) as well as a lack of replicates in previous investigations. Given the diverse regulatory roles of miRNAs in multiple pathways, it is possible that the alterations and regulatory functions of miRNAs may vary depending on the disease stage. Therefore, changes in the expression levels of miRNAs may not necessarily be persistent during the course of disease progression.

Bioinformatics analysis suggested that *miR-21*, *miR-25*, *miR-146a*, and *miR-181a* regulate multiple genes in pathways associated with immunity and inflammatory responses related to the pathogenesis of autoimmune diabetes, such as TNF, Toll-like receptor, MAPK, Fc epsilon RI, thyroid hormone, osteoclast differentiation, T-cell receptor, NOD-like receptor, and AGE-RAGE signaling pathways. Additionally, multiple signaling pathways associated with metabolism and diabetes/hyperglycemia were enriched with target genes of these four miRNAs, such as sphingolipid, cGMP-PKG, FoxO, AMPK, Rap1, and insulin signaling pathways. Furthermore, multiple signaling pathways have been reported to participate in the development of diabetic complications. The AGE-RAGE signaling pathway was recently reported to promote diabetes-mediated vascular calcification via increasing oxidative stress [32]. The axon guidance signaling pathway was previously shown to be involved in the development of diabetic nephropathy and retinopathy [33, 34]. Additionally, the Rap1 signaling pathway is inhibited by hyperglycemia, resulting in tubular injury in individuals with diabetic nephropathy [35].

Multiple logistic regression analysis identified *miR-25*, *miR-146a*, and FCP as independent predictors for autoimmune diabetes, and a nomogram constructed based on these data exhibited favorable performance in estimation of autoimmune diabetes, suggesting the potential applications of these targets as circulating biomarkers for autoimmune diabetes. However, because most patients with autoimmune diabetes included in this study had a disease duration of several years, we did not directly demonstrate the predictive roles of *miR-25* and *miR-146a* for autoimmune diabetes. Therefore, further studies comparing individuals at risk for autoimmune diabetes with healthy controls are necessary to validate the predictive value of *miR-25* and *miR-146a*.

There were several limitations to this study. First, the results of the current study differed from those of previous publications, and we speculate that differences in study populations and methods used to measure miRNAs may explain these inconsistencies, which could lead to the failure of direct comparison with the results of the previous study. Second, the restricted generalizability of the results and decreased statistical power need to be considered, given the relatively small sample size. Third, to the best of our knowledge, for the first time, we found similar decreases in *miR-146a* and *miR-181a* levels in patients with LADA, suggesting the possibility that LADA and T1D share similar pathogenic mechanisms. However, further studies are required to validate these results. Lastly, our study was a cross-sectional observational study; thus, we were able to identify only associations, not causal relationships of the four miRNAs with autoimmune diabetes.

## 5. Conclusions

In summary, our findings suggested that serum *miR-21*, *miR-25*, *miR-146a*, and *miR-181a* were significantly downregulated in autoimmune diabetes and may be associated with regulation of the pathogenesis of autoimmune diabetes from various aspects, including autoimmunity, inflammation responses, pancreatic *β*-cell apoptosis, and impaired insulin secretion. Furthermore, serum *miR-25* and *miR-146a* may serve as independent predictors for estimation of autoimmune diabetes. These findings could have relevance for clinical practice and suggest that these miRNAs could serve as novel circulating biomarkers of the pathogenesis of autoimmune diabetes. Further investigations are necessary to illuminate their functional roles in the pathogenesis of autoimmune diabetes.

## Figures and Tables

**Table 1:** Clinical characteristics of study population.

		T1D (*n* = 29)	LADA (*n* = 16)	T2D (*n* = 31)	Nondiabetic individuals (*n* = 19)
Sex (male/female)		10/19	10/6	19/12	15/4
Age (years)		24.0 ± 9.1^abc^	51.9 ± 9.5^ad^	44.9 ± 7.7^ad^	30.3 ± 5.0^abc^
Age of onset (years)		20.8 ± 10.0^bc^	46.3 ± 8.7^a^	41.7 ± 6.5^a^	N/A
Duration (years)		3.4 ± 4.2	5.6 ± 5.0	3.2 ± 2.4	N/A
BMI (kg/m^2^)		21.1 ± 3.9	21.9 ± 2.7	23.1 ± 2.6	23.2 ± 2.9
HbA1c (%)		8.7 ± 2.3^d^	8.4 ± 1.5^cd^	7.2 ± 1.8^bd^	5.1 ± 0.2^abc^
FCP (ng/dL)		0.46 ± 0.63^cd^	0.16 ± 0.20^cd^	0.63 ± 0.56^ab^	1.24 ± 0.31^ab^
2hCP (ng/dL)		1.16 ± 1.86^cd^	0.35 ± 0.49^cd^	5.02 ± 2.79^ab^	3.72 ± 1.24^ab^
TC (mmol/L)		4.80 ± 1.69^c^	4.56 ± 1.19^c^	5.53 ± 0.91^abd^	5.00 ± 1.35^c^
TG (mmol/L)		1.10 ± 0.83	0.82 ± 0.65^c^	1.82 ± 1.15^bd^	1.10 ± 0.64^c^
HDL-C (mmol/L)		1.43 ± 0.40	1.51 ± 0.47	1.20 ± 0.23	1.34 ± 0.27
LDL-C (mmol/L)		2.78 ± 1.35	2.45 ± 0.79	3.10 ± 0.68^d^	2.46 ± 1.08^c^
GAD titers (IU/mL)		260.8 ± 457.0^bcd^	354.7 ± 363.1^acd^	0^ab^	0^ab^
IA-2A titers (IU/mL)		688.8 ± 1322.5^bcd^	288.6 ± 995.0^bcd^	0^ab^	0^ab^
ICA titers (IU/mL)		6.9 ± 10.0^cd^	5.0 ± 6.3^cd^	0^ab^	0^ab^

Note: a, compared with T1D, *p* < 0.05; b, compared with LADA, *p* < 0.05; c, compared with T2D, *p* < 0.05; d, compared with nondiabetic individuals, *p* < 0.05. Abbreviations: T1D, type 1 diabetes; LADA, latent autoimmune diabetes of adults; T2D, type 2 diabetes; BMI, body mass index; HbA1c, glycosylated hemoglobin; FCP, fasting C-peptide; 2hCP, 2-h postprandial C-peptide; TC, total cholesterol; TG, total triglyceride; HDL-C, high-density lipoprotein cholesterol; LDL-C, low-density lipoprotein cholesterol; GADA, glutamic acid decarboxylase antibody; IA-2A, protein tyrosine phosphatase antibody; ICA, islet cell antibody.

**Table 2:** Association of serum levels of *miR-21*, *miR-25*, *miR-146a*, and *miR-181a* with clinical parameters in autoimmune diabetes.

	*miR-21*	*miR-25*	*miR-146a*	*miR-181a*
Sex	0.024	−0.231	−0.150	0.064
Age	0.269	0.576^*∗∗*^	0.260	0.016
BMI	−0.069	0.296^*∗*^	0.046	−0.151
Age of onset	0.192	0.483^*∗∗*^	0.249	−0.026
Duration	0.156	0.265	−0.025	−0.100
HbA1c	0.079	−0.115	0.147	0.175
FCP	−0.106	−0.284	0.011	0.079
2hCP	−0.173	−0.132	−0.006	0.045
TC	0.021	−0.159	0.059	−0.020
TG	−0.093	−0.315	0.009	−0.100
HDL-C	0.209	−0.034	0.026	−0.095
LDL-C	−0.018	−0.239	0.020	0.053
GADA titers	0.006	−0.069	0.235	0.039
ICA titers	−0.028	−0.152	−0.083	0.118
IA-2A titers	−0.181	−0.224	−0.077	0.116
Number of positive antibodies	0.157	−0.058	0.125	0.166

Note: ^*∗*^*p* < 0.05; ^*∗∗*^*p* < 0.01. Abbreviations: T1D, type 1 diabetes; LADA, latent autoimmune diabetes of adults; T2D, type 2 diabetes; BMI, body mass index; HbA1c, glycosylated hemoglobin; FCP, fasting C-peptide; 2hCP, 2-h postprandial C-peptide; TC, total cholesterol; TG, total triglyceride; HDL-C, high-density lipoprotein cholesterol; LDL-C, low-density lipoprotein cholesterol; GADA, glutamic acid decarboxylase antibody; IA-2A, protein tyrosine phosphatase antibody; ICA, islet cell antibody.

## Data Availability

The SPSS Statistics data used to support the findings of this study are available from the corresponding author upon request.

## Supplementary Materials

Supplementary Materials Table 1: KEGG pathways enriched with genes targeted by the 4 miRNAs with *p* < 0.01. Figure 1: forest plot based on multiple logistic regression analysis.
