# Supplementary material for: Decreased Serum microRNA-21, microRNA-25, microRNA-146a, and microRNA-181a in Autoimmune Diabetes: Potential Biomarkers for Diagnosis and Possible Involvement in Pathogenesis
Source: Int J Endocrinol. 2019 Sep 9;2019:8406438. doi: 10.1155/2019/8406438 (PMC6754900; doi:10.1155/2019/8406438)
Supplement: Supplementary Materials — Table 1: KEGG pathways enriched with genes targeted by the 4 miRNAs with p < 0.01. Figure 1: forest plot based on multiple logistic regression analysis. [file 8406438.f1.docx]

Supplemental table 1. KEGG pathways enriched with genes targeted by the 4 miRNAs with p<0.01.

|  |  | Number of genes in pathway | | |
| --- | --- | --- | --- | --- |
| miRNA(s) | KEGG pathway | Total | Targeted | *P* |
| All 4 miRNAs | TNF signaling pathway | 110 | 12 | 1.54E-04 |
|  | AGE-RAGE signaling pathway in diabetic complications | 101 | 11 | 2.21E-04 |
|  | mTOR signaling pathway | 154 | 13 | 3.46E-04 |
|  | Sphingolipid signaling pathway | 121 | 11 | 5.99E-04 |
|  | Chagas disease (American trypanosomiasis) | 104 | 10 | 7.78E-04 |
|  | Influenza A | 176 | 13 | 7.78E-04 |
|  | Toll-like receptor signaling pathway | 106 | 10 | 8.31E-04 |
|  | Type II diabetes mellitus | 48 | 7 | 8.90E-04 |
|  | RIG-I-like receptor signaling pathway | 70 | 8 | 1.12E-03 |
|  | cGMP-PKG signaling pathway | 167 | 12 | 1.31E-03 |
|  | Insulin resistance | 109 | 9 | 2.82E-03 |
|  | Tight junction | 139 | 10 | 3.27E-03 |
|  | Hepatitis B | 146 | 10 | 4.04E-03 |
|  | Epstein-Barr virus infection | 204 | 12 | 4.04E-03 |
| miR-21,miR-25 and miR-146a | Longevity regulating pathway | 94 | 8 | 4.00E-03 |
| miR-21,miR-25 and miR-181a | MAPK signaling pathway | 255 | 23 | 5.17E-07 |
|  | FoxO signaling pathway | 134 | 13 | 1.57E-04 |
|  | HTLV-I infection | 259 | 18 | 1.61E-04 |
|  | Fc epsilon RI signaling pathway | 68 | 8 | 1.01E-03 |
|  | GnRH signaling pathway | 91 | 9 | 1.12E-03 |
|  | Dorso-ventral axis formation | 28 | 5 | 2.82E-03 |
|  | cAMP signaling pathway | 199 | 12 | 3.97E-03 |
|  | Thyroid hormone signaling pathway | 118 | 9 | 3.99E-03 |
|  | ErbB signaling pathway | 88 | 7 | 8.19E-03 |
| miR-21,miR-146a and miR-181a | Neurotrophin signaling pathway | 120 | 15 | 3.41E-06 |
|  | Axon guidance | 176 | 11 | 4.04E-03 |
|  | Chemokine signaling pathway | 187 | 14 | 4.77E-04 |
|  | Toxoplasmosis | 119 | 10 | 1.35E-03 |
|  | Osteoclast differentiation | 132 | 10 | 2.74E-03 |
|  | Focal adhesion | 203 | 12 | 4.04E-03 |
|  | Tuberculosis | 179 | 11 | 4.34E-03 |
|  | AMPK signaling pathway | 125 | 9 | 4.34E-03 |
|  | Wnt signaling pathway | 143 | 9 | 8.77E-03 |
| miR-25,miR-146a and miR-181a | 0 |  |  |  |
| miR-21,miR-25 | Hippo signaling pathway | 154 | 11 | 2.32E-03 |
| miR-21,miR-181a | Protein processing in endoplasmic reticulum | 166 | 15 | 1.06E-04 |
|  | Endocrine resistance | 97 | 10 | 5.52E-04 |
|  | Prolactin signaling pathway | 72 | 8 | 1.25E-03 |
|  | Amyotrophic lateral sclerosis (ALS) | 51 | 6 | 4.04E-03 |
|  | Platelet activation | 122 | 9 | 4.04E-03 |
|  | Rap1 signaling pathway | 211 | 12 | 4.53E-03 |
|  | Oxytocin signaling pathway | 158 | 10 | 5.30E-03 |
|  | T cell receptor signaling pathway | 105 | 8 | 5.39E-03 |
|  | Hypertrophic cardiomyopathy (HCM) | 83 | 7 | 6.21E-03 |
|  | Insulin signaling pathway | 139 | 9 | 7.58E-03 |
| miR-146a,miR-181a | NOD-like receptor signaling pathway | 57 | 6 | 5.20E-03 |
| miR-21,miR-146a | 0 |  |  |  |
| miR-25,miR-146a | 0 |  |  |  |
| miR-25,miR-181a | 0 |  |  |  |
| miR-21 | Adrenergic signaling in cardiomyocytes | 149 | 10 | 4.04E-03 |
| miR-25 | Hippo signaling pathway -multiple species | 29 | 5 | 3.04E-03 |
| miR-146a | 0 |  |  |  |
| miR-181a | Primary immunodeficiency | 37 | 6 | 1.32E-03 |
|  | Long-term potentiation | 66 | 7 | 3.06E-03 |
|  | Dopaminergic synapse | 130 | 9 | 5.29E-03 |
|  | Dilated cardiomyopathy | 90 | 7 | 8.90E-03 |

Pathways related to malignancy were eliminated. Note: KEGG, Kyoto Encyclopedia of Genes and Genomes.

Supplemental Figure 1. Forest plot based on multiple logistic regression analysis. Abbreviation：OR, odds ratio; FCP, fasting C peptide.
